# Sex differences in CRT device implantation rates, efficacy, and complications following implantation: protocol for a systematic review and meta-analysis of cohort studies

**DOI:** 10.1186/s13643-021-01746-x

**Published:** 2021-07-23

**Authors:** Omar Dewidar, David Birnie, Irina Podinic, Vivian Welch, George A. Wells

**Affiliations:** 1 School of Epidemiology and Public Health, University of Ottawa, 600 Peter Morand Crescent, Ottawa, Ontario K1G 5Z3 Canada; 2 Bruyère Research Institute, University of Ottawa, 85 Primrose Ave, Ottawa, Ontario K1R 6M1 Canada; 3 Division of Cardiology, Department of Medicine, University of Ottawa Heart Institute, 40 Ruskin St, Ottawa, Ontario K1Y 4W7 Canada; 4 Cardiovascular Research Methods Centre, University of Ottawa Heart Institute, 40 Ruskin St, Ottawa, Ontario K1Y 4W7 Canada

**Keywords:** Cardiac resynchronization therapy, Cardiac implantable electronic device, Efficacy, Complications, Implantation, Cohort, Sex differences, Non-randomized studies, Systematic review

## Abstract

**Introduction:**

There is abundant evidence for sex differences in the diagnosis, implantation, and outcomes for cardiac resynchronization therapy (CRT) devices. Controversial data suggesting women are less likely to receive the device regardless of the greater benefit. The aim of this review is to assess sex differences in the implantation rate, clinical effectiveness, and safety of patients receiving CRT devices.

**Methods:**

We will conduct a systematic literature search of MEDLINE, Embase, and Web of Science to identify cohort studies that meet our eligibility criteria. Title and full text screening will be conducted in duplicate independently. Eligible studies report clinical effectiveness or safety of patients receiving CRT device while providing sex-disaggregated data. Implantation rate will be extracted from the baseline characteristics tables of the studies. The effectiveness outcomes include the following: all-cause death, hospitalization, peak oxygen consumption (pVO_2_), quality of life (QoL), 6-min walk test, NYHA class reduction, LVEF, and heart failure hospitalization. The complication outcomes include the following: contrast-induced nephropathy, pneumothorax, pocket-related hematoma, pericardial tamponade, phrenic nerve stimulation, device infection, death, pulmonary edema, electrical storm, cardiogenic shock, and hypotension requiring resuscitation. Description of included studies will be reported in detail and outcomes will be meta-analyzed and presented using forest plots when feasible. Risk of bias will be assessed using the Newcastle-Ottawa Scale (NOS) by two review authors independently. GRADE approach will be used to assess the certainty of evidence.

**Discussion:**

The aim of this review is to determine the presence of differences in CRT implantation between women and men as well as differences in clinical effectiveness and safety of CRT after device implantation. Results from this systematic review will provide important insights into sex differences in CRT devices that could contribute to the development of sex-specific recommendations and inform policy.

**Systematic review registration:**

PROSPERO CRD42020204804

## Background

There are substantial behavioral and biological differences between men and women which affect the manifestation, epidemiology, and pathophysiology of diseases and potential therapies [1]. Sex and gender affect an array of diseases including cardiovascular disease [2]. Heart failure (HF), one of the most prevalent cardiovascular diseases, is the leading cause of cardiovascular morbidity and mortality in Canada and the world and also differentially impacts men and women in terms of risk and outcomes [3, 4]. Biological differences cause women to more frequently experience heart failure with atypical symptoms unlike men which results in underdiagnosis [5, 6].

Cardiac resynchronization therapy (CRT), also known as biventricular pacing is the latest cardiac implantable electronic device (CIED) aimed to reduce symptoms for HF and improve ventricular function heart. This device exists in two types: CRT-pacemaker (CRT-P), which synchronizes the heart beating pattern through improving the pumping action the lower ventricles, and CRT-defibrillator (CRT-D), which in addition to synchronizing heart rhythm, can detect and treat sudden cardiac death (SCD) [7]. International cardiovascular guidelines have declared CRT devices as the highest recommendation (class 1 indication) for patients with left ventricular ejection fraction (LVEF) ≤ 35%, New York Heart Association (NYHA) heart failure classification 2, 3, or 4 symptoms (scale corresponding to patient’s inability to conduct physical activity), left bundle block (LBBB) with a QRS duration ≥ 150 ms, and sinus rhythm [8].

Sex differences are apparent in heart failure pathophysiology and etiology; however, the mechanisms remain not well understood [9]. An in-depth review of observational studies and trials assessing sex differences in heart failure therapies [10] reports controversial findings of underutilization of CRT devices in women compared to men [11–16] even though there is greater clinical benefit in women and diverse findings for adverse events [14, 17, 18]. Evidence from an individual participant data (IPD) network meta-analysis (MA) of 3 trials [19–21] (n = 4076) suggests that women with LBBB and QRS of 150 ms or longer benefited from CRT-D more than men, with 76% reduction in heart failure [22]. However, underrepresentation of women (only 20% of the total population) and variety in follow-up times (1.1, 2.2, and 4.7 years across 3 trials) proposes cautious interpretation of the results.

A systematic review of randomized and non-randomized (NRS) CRT studies (n = 183) suggests that women with CRT devices tend to have better LVEF reduction < 15% compared to men [23]. However, this review was conducted in 2015 and is of low quality as per AMSTAR 2 tool [24], with single database search (PubMed) in combination with study restriction via text search and absence of risk of bias (RoB) assessment and inappropriate pooling of studies for MA among other issues [25].

Most of the previous SRs and MAs have focused on RCTs. However, to better interpret the effectiveness and safety of CRT devices across sex in the usual clinical setting, we will restrict to cohort studies as the results tend to be more generalizable [26], implantation rates will reflect real-world setting, overcome the issue of female underrepresentation in clinical trials [27], and spurious findings from post hoc subgroup analyses [28].

The objectives of this review are to formally assess sex differences in the (1) implantation rate, (2) clinical effectiveness, and (3) safety for patients receiving CRT devices.

## Methods

This protocol was developed a priori to conducting the study. Any deviations will be reported in all related publications and the PROSPERO record (CRD42020204804) will be adapted accordingly. This systematic review protocol was developed using guidance from the Cochrane Handbook for Systematic Review of Interventions [29] and the Preferred Reporting Items for Systematic Review and Meta-Analysis Protocols (PRISMA-P) [30].

### Eligibility criteria

#### Participants

This review will target patients ≥ 18 years eligible for de novo implantation of CRT conforming with international guidelines [8]. Studies in which the population underwent device replacement will not be included; however, we will consider patients that received upgrade from ICD to CRT-D.

#### Interventions

The intervention of the included studies will be the CRT device in addition to follow-up from time of implantation of CRT in accordance to guidelines [31, 32]. Follow-up methods can be conducted virtually or by in clinic visits.

#### Outcomes

Studies must report outcomes disaggregated by sex or compare outcomes across sex.

##### Implantation rate

Implantation rate will correspond to the frequency of device implantations for each sex as a proportion of the total number of implantations.

##### Effectiveness

The definitions for the effectiveness outcomes are reported in Appendix 1 and were selected based on reporting in international clinical guidelines. Clinical efficacy outcomes include the following: all-cause mortality, hospitalization, peak oxygen consumption (pVO_2_), quality of life (QoL), 6-min walk test, NYHA class reduction, LVEF, and heart failure hospitalization (defined in Appendix 1).

##### Complications

We plan to assess both clinical and mechanical complications as categorized by Krahn et al. [33] where mechanical complications stem from mechanical effects of the surgery, while clinical complications arise from new or worsening comorbidities. All the complications to be assessed will be “major” as they will require intervention to provide therapeutic relief [34]. Complications were classified short term if they occurred within 2 months of implantation and long term if they emerged thereafter [35]. We will assess the emergence of the following device complications (defined in Appendix 2): contrast-induced nephropathy, pneumothorax, pocket-related hematoma, pericardial tamponade, phrenic nerve stimulation, device infection, death, pulmonary edema, electrical storm, cardiogenic shock, and hypotension requiring resuscitation.

#### Study designs

We will include in our review cohort studies (prospective, retrospective). We will exclude randomized studies, case reports, case series, review articles, cross-sectional studies, surveys, qualitative or interview/focus group studies, editorials, letters, and commentaries.

#### Setting

No restrictions will be imposed on the type of setting.

#### Language

No language restriction will be applied.

### Information sources

The search was conducted from the date January 1, 2000, to June 12, 2020, of the following databases: MEDLINE, Embase, and Web of Sciences. To ensure a thorough search, we will review the reference list of relevant systematic reviews for eligible studies.

### Search strategy

With the assistance of an information specialist, we developed a comprehensive search strategy. The search strategy includes a combination of Medical Subject Headings (MeSH) and indexed terms and database-specific terms. Detailed description of the search strategies can be found in Appendix 3.

Keywords used to develop this search are variations of the following terms: “Cardiac Implantable Electronic Device” or “Cardiac Resynchronization Therapy” or “Defibrillators” or “Implantable cardioverter defibrillator” or “heart failure” or “Cardiac failure” or “Biventricular Pacemaker”. Due to the large number of studies retrieved (> 20,000), we will use the most up-to-date cohort study search filter developed by the Inter InterTASC Information Specialists’ Sub-Group (ISSG) [36] to obtain observational studies. We also added variants of the terms: “Registry”, “Prospective” and “Retrospective” for a more comprehensive search. To further narrow our search, we will use a validated heart failure search filter that retrieves studies focused on device therapy in heart failure context [37].

### Study selection and screening process

Study selection will be conducted in a blinded fashion, by two independent reviews using Covidence program [38]. Screening at title and abstract stage and full text stages will be completed in duplicate. Discrepancies will be resolved by discussion between reviewers followed by consensus, or a third reviewer. We will prepare a PRISMA study flow chart. Studies that assess sex differences in outcomes for patients receiving CRT devices will be selected in duplicate, independently. We plan on capturing all outcomes reported in the studies that relate to the clinical effectiveness of the device, implantation rate across sex, and adverse events related to complications post implantation. ICD studies with > 50% CRT-D will be considered eligible and extracted. Studies with > 75% primary prevention will be extracted, and other studies will be listed with reason for exclusion (e.g., secondary prevention).

### Data collection

Data extraction will be conducted by two independent reviewers using a pretested extraction form. Conflicts will be resolved by consensus between the two reviewers or by a third reviewer. The extraction form will be developed in collaboration with a content expert based on the Data Extraction Template for Cochrane Reviews [39]. The form will be pilot tested with a random sample of 5 studies and further developed accordingly. The extraction process will be facilitated using Microsoft Excel forms customized with extraction criteria.

### Data items

The extraction form will be developed in collaboration with a content expert, and pilot tested with a random sample of 5 studies, and further developed accordingly.

Reviewers will extract data on study characteristics (name of author, publication date, journal, funding, and conflict of interest), study methodology (objectives, target population, recruitment and sampling procedures, setting), participant information (baseline characteristics, device type, disease severity classification, sample size), and outcomes (definitions, time of measurement, and results) (Appendix 1).

### Risk of bias and quality of assessment of individual studies

We will be assessing non-randomized studies; therefore, we will be using the Newcastle-Ottawa Scale (NOS) tool to assess risk of bias [40]. Scale items assess selection of cases and controls, comparability of cases and controls, and outcome follow-up. Judgments will be made by two independent reviewers and disagreements will be resolved through discussion.

### Synthesis of results

We will meta-analyze quantitative data when appropriate. We will use I^2^ statistic to assess heterogeneity. An I^2^ value greater than 75% would indicate high heterogeneity. An I^2^ between 50 and 75% indicates moderate heterogeneity and calls for investigation via post hoc subgroup analysis [41]. We will conduct meta-analysis using random effects methodology using Revman [42]. When meta-analysis is inappropriate due to comparative diversity, results will be narratively reported while providing effect sizes and confidence intervals. Individual and pooled analyses will be presented using tables and forest plots, respectively.

Interventions that involve implantation of CRT-P and CRT-D will be treated separately. All the extracted outcomes will be analyzed separately. Categorical outcomes will be assessed using risk ratios (95% confidence intervals (CI)) and continuous outcomes will be analyzed using mean differences (95% CI) [43].

An intention to treat analysis method will be adopted. We will record how authors dealt with missing data and sensitivity analysis will be used to investigate the effect of missing data on the overall results. Adjusted results reported in the included studies as effect measures will be used in the analyses accordingly.

#### Subgroup analysis

Where feasible and appropriate, we will conduct exploratory subgroups to investigate the impact of factors on outcomes. We plan on conducting a priori subgroup analysis for the following criteria:

- Age (< 65, ≥ 65)
- Type of device (CRT-D, CRT-P)
- Disease severity (NYHA class I–II, NYHA class III–IV)
- QRS morphology (LBBB, non-LBBB)
- Heart failure etiology (ischemic, non-ischemic)

#### Sensitivity analysis

Sensitivity analysis may be considered with respect to composition of the participants or definitions of outcomes to assess the robustness of the performed meta-analysis. We will inspect publication bias using funnel plots in the case of 10 or more studies are included in our review [44]. We will display the meta-analyzed results using forest plots.

### Strength of evidence assessment

We will use the GRADE framework to rate the certainty of the evidence of intervention effects [45]. GRADE assessment will be conducted in duplicate by two reviewers, independently. Discrepancies will be resolved by discussion or a third reviewer. Summarized results will be presented as summary of findings tables [45].

### Discussion

The aim of this review is to determine the presence of differences in CRT implantation between women and men as well as differences in efficacy and safety of CRT post device implantation. We anticipate our systematic review will provide important insights into sex differences in CRT devices that could eventually lead to develop sex-specific recommendations and inform policy.

A potential limitation to this review is the inability to retrieve disaggregated data for some of the included studies that do not report data in the manuscript. We will not attempt to contact authors for sex-specific data as we predict a high yield of eligible studies and anticipate little reward with contacting as it is a time-consuming process. However, our pilot search has identified several studies with disaggregated data so this will not impede the ability to conduct quantitative meta-analysis. We also acknowledge that implementing our search filters restricting by study design and heart failure topic may result in missing eligible studies, but we plan on hand searching eligible systematic reviews to ensure a complete search.

We acknowledge that acquiring measures of effect from NRS is not always reliable due to confounding and other biases. However, several reviews have indicated that the results of NRS correspond generally with the results from trials when properly designed with appropriate accounting for covariates [46–50], providing accurate estimates of effects in real-world practice setting. In addition, ethical issues arise with conducting RCTs primarily aimed at assessing therapies that are already implemented in health policy due to equipoise. In general, RCTs for surgical treatments are of poor methodological quality due to difficulties in randomization, incapability of blinding, and small sample sizes [51, 52]. Therefore, treatment information is primarily obtained from NRS evidence [53, 54]. This paradigm remains valid for cardiac surgery studies as there is a significant reduction in the number of RCTs over the years [55].

Furthermore, the absence of participant restrictions in observational studies makes them well suited to study effects on health inequities which are differences in health that are avoidable and unfair [56]. Patient-physician gender, patient preference, and gender bias impact the choice and efficacy of cardiovascular interventions [57–59]. The use of RCTs to investigate this question will be inadequate since researchers manipulate the enrolment of participants.

We are aware that observational studies cannot replace RCTs as they provide high internal validity and comparability, but RCTs cannot be axiomatic. Results from both study designs can complement each other to fill gaps in clinical knowledge and contribute to decision making.

## Data Availability

Not applicable.

## Appendix 1

**Table 1:** Definitions of efficacy outcomes

Efficacy outcomes	Definitions
6-min walk test	Treadmill walking test to test exercise capacity in patients with chronic heart failure [60].
NYHA class reduction	Estimation of NYHA functional class [61] within 6 months after randomization [62]
LVEF improvement	Measured using 2-dimenionsonal Doppler-flow echocardiography [63] or used LV volume reduction
Peak oxygen consumption (pVO_2_)	Measurement of peak oxygen consumption per unit time at anaerobic threshold. The improvement will be assessed from baseline to 6 months [64]
Hospitalization	NA
All-cause mortality	NA
Quality of life	Questionnaire developed to assess patients perception on the impact of heart failure on their daily lifestyle [65]
Heart failure hospitalization	Includes patients that were admitted to any health facility for the treatment of heart failure symptoms for more than 24 h [66]
Composite response endpoint	**Worsened**: Patient death, worsening of heart failure resulting in hospitalization, therapeutic response is considered insufficient, worsening in NYHA class relative to previous observation or moderate-marked worsening of patient global assessment score at LOCF (last observation carried forward). **Improved**: Patient has not worsened (as previously described) and shows improvement in NYHA class at LOCF and/or moderate-marked improvement in patient global assessment score at LOCF. **Unchanged**: No indications of worsened nor improved health of patients [62].
All-cause mortality composite outcomes	All-cause mortality in combination with any other outcomes

## Appendix 2

**Table 2:** Definitions of safety outcomes

Short term or long term	Complications	Definition
Mechanical		
Long term	Lead-related complications (i.e., dislodgement, lead malposition)	Presence of lead malfunction requiring reoperation [67]
Short term or long term	Device infection	Hospitalization for proven CIED infection within 1 year of implantation. Infection is categorized into pocket infection, bloodstream infection, and endocarditis [68].
Short term	Contrast-induced nephropath	Contrast nephropathy was defined as an increase in serum creatinine of 25% or greater within 48 h after contrast administration. Contrast nephropathy was defined as an increase in serum creatinine of 25% or greater within 48 h after contrast administration. Contrast nephropathy was defined as an increase in serum creatinine of 25% or greater within 48 h after contrast administration. Contrast nephropathy was defined as an increase in serum creatinine of 25% or greater within 48 h after contrast administration which would lead to dialysis [69].
Short term	Pneumothorax (related to venous access)	Complications while obtaining venous access during the index hospitalization including the absence of lung markings over the lung field ipsilateral to the PM pocket assessed from the predischarge X-ray [70].
Short term	Pocket-related Hematoma	Hematoma requiring further surgery, resulting in prolongation of hospitalization, or requiring interruption of oral anticoagulation therapy. Prolongation of hospitalization was defined as extended hospitalization or rehospitalization for at least 24 h after the index surgical procedure, primarily due to the hematoma [71].
Short term	Pericardial tamponade	Slow or rapid compression of the heart due to the pericardial accumulation of fluid, pus, blood, clots, or gas, as a result of effusion, trauma, or rupture of the heart [72].
Short term	Phrenic nerve stimulation requiring reoperation	PS tested during follow-up of patients starting from maximum pacing system analyzer output, 10 V at 1.5 ms followed by a stepping down protocol. In the event of PS occurrence, its threshold is measured in all the possible pacing configurations and compared with LV pacing threshold to ensure the feasibility of biventricular stimulation. PS disappearance was defined as absence of muscular stimulation over a 20-min observation period during respiratory changes (deep breath); LV threshold was defined as 100% stimulation during the same respiratory changes and requires reoperation [73].
Clinical		
Short term	Death	Clinical death was considered to be when spontaneous respirations ceased, and pulse and blood pressure disappeared [74].. Cause of death of patients before their first outpatient visit must be established by reviewing patient charts to identify relation to procedure [75].
Short term	Electrical storm	Electric storm in patients with CRT-D is defined as ≥ 3 adequate detections of VT and/or VF in 24 h terminated with ATP or high-voltage therapy (HVT), or untreated sustained VT recorded in the monitoring zone over 1 week after the implantation [76–79].
Short term	Pulmonary edema	NA
Short term	Cardiogenic shock	Defined as hypotension (SBP, 90 mmHg) despite adequate filling status with signs of hypoperfusion [80].
Short term	Hypotension requiring resuscitation	NA

## Appendix 3

Detailed search strategy

MEDLINE:

1. CIED or (Cardiac adj2 implant* adj2 electronic* adj2 device?)).ti,ab.
2. defibrillators, implantable/ or exp pacemaker, artificial/
3. ((biventricular adj2 pacemaker*) or pace-maker*).ti,ab.
4. ((resynch* or re-synch*) adj3 (cardiac or therap* or treatment* or device*)).ti,ab.
5. (cardioconver* or (cardio adj conver*)).ti,ab.
6. 1 or 2 or 3 or 4 or 5
7. exp cohort studies/
8. cohort$.tw.
9. controlled clinical trial.pt.
10. epidemiologic methods/
11. limit 10 to yr=1971-1988
12. or/7-9,11
13. (registr* or Prospective* or Retrospective*).mp.
14. 12 or 13
15. heart failure.mp.
16. ventricular dysfunction, left.sh.
17. cardiomyopathy.mp.
18. left ventricular ejection fraction.mp.
19. or/13-16
20. 6 and 14 and 19
21. limit 20 to yr=“2000 -Current”

Embase:

1. CIED or (Cardiac adj2 implant* adj2 electronic* adj2 device?)).ti,ab.
2. cardiac implantable electronic device/ or exp cardiac resynchronization therapy device/
3. exp biventricular implantable cardioverter defibrillator/ or exp cardiac resynchronization therapy defibrillator/ or exp dual chamber implantable cardioverter defibrillator/
4. exp defibrillator pacemaker/
5. ((biventricular adj2 pacemaker*) or pace-maker*).ti,ab.
6. ((resynch* or re-synch*) adj3 (cardiac or therap* or treatment* or device*)).ti,ab.
7. (cardioconver* or (cardio adj conver*)).ti,ab.
8. 1 or 2 or 3 or 4 or 5 or 6 or 7
9. exp cohort analysis/
10. exp longitudinal study/
11. exp prospective study/
12. exp follow up/
13. cohort$.tw.
14. or/9-13
15. (registr* or Prospective* or Retrospective*).mp.
16. 14 or 15
17. heart failure.mp.
18. ventricular dysfunction, left.sh.
19. cardiomyopathy.mp.
20. left ventricular ejection fraction.mp.
21. or/15-18
22. 8 and 16 and 21
23. limit 22 to yr=“2000 -Current”
24. (conference abstract or conference review).pt.
25. 23 not 24

Web of Science

1. TS=(CIED or (Cardiac NEAR/2 implant* NEAR/2 electronic* NEAR/2 device*))
2. TS=((biventricular NEAR/2 pacemaker*) or pace-maker*)
3. TS=((resynch* or re-synch*) NEAR/3 (cardiac or therap* or treatment* or device*))
4. TS=(cardioconver* or (cardio adj conver*))
5. 1 or 2 or 3 or 4
6. TS=“heart failure”
7. TS=(left ventric* NEAR/2 “dysfunction”)
8. TS=(left ventric* NEAR/2 “ejection fraction”)
9. 6 or 7 or 8 or 9
10. 10 and 5
11. TS=Cohort
12. TS=longitudinal*
13. TS=prospective*
14. TS=“follow up”
15. TS = registr*
16. TS = Retrospective*
17. 11 or 12 or 13 or 14 or 15 or 16
18. 18 and 11

## Abbreviations

HF: Heart failure
CRT: Cardiac resynchronization therapy
CIED: Cardiac implantable electronic device
CRT-P: Cardiac resynchronization therapy-pacemaker
CRT-D: Cardiac resynchronization therapy-defibrillator
LVEF: Left ventricular ejection fraction
LBBB: Left bundle block branch
PRISMA: Preferred Reporting Items for Systematic Review and Meta-Analysis
PRISMA-P: Preferred Reporting Items for Systematic Review and Meta-Analysis Protocols
QoL: Quality of life
